# A novel score for predicting falls in community-dwelling older people: a derivation and validation study

**DOI:** 10.1186/s12877-024-05064-4

**Published:** 2024-06-04

**Authors:** Ming Zhou, Gongzi Zhang, Na Wang, Tianshu Zhao, Yangxiaoxue Liu, Yuhan Geng, Jiali Zhang, Ning Wang, Nan Peng, Liping Huang

**Affiliations:** 1 Department of Rehabilitation Medicine, The First Medical Center of Chinese PLA General Hospital, Beijing, China; 2 Department of Rehabilitation Medicine, The Second Medical Center & National Clinical Research Center for Geriatric Diseases, Chinese PLA General Hospital, Beijing, China; 3 Medical School of Chinese PLA, Beijing, China

**Keywords:** Accidental falls, Older adults, Risk assessment, Fall prediction

## Abstract

**Background:**

Early detection of patients at risk of falling is crucial. This study was designed to develop and internally validate a novel risk score to classify patients at risk of falls.

**Methods:**

A total of 334 older people from a fall clinic in a medical center were selected. Least absolute shrinkage and selection operator (LASSO) regression was used to minimize the potential concatenation of variables measured from the same patient and the overfitting of variables. A logistic regression model for 1-year fall prediction was developed for the entire dataset using newly identified relevant variables. Model performance was evaluated using the bootstrap method, which included measures of overall predictive performance, discrimination, and calibration. To streamline the assessment process, a scoring system for predicting 1-year fall risk was created.

**Results:**

We developed a new model for predicting 1-year falls, which included the FRQ-Q1, FRQ-Q3, and single-leg standing time (left foot). After internal validation, the model showed good discrimination (C statistic, 0.803 [95% CI 0.749–0.857]) and overall accuracy (Brier score, 0.146). Compared to another model that used the total FRQ score instead, the new model showed better continuous net reclassification improvement (NRI) [0.468 (0.314–0.622), *P* < 0.01], categorical NRI [0.507 (0.291–0.724), *P* < 0.01; cutoff: 0.200–0.800], and integrated discrimination [0.205 (0.147–0.262), *P* < 0.01]. The variables in the new model were subsequently incorporated into a risk score. The discriminatory ability of the scoring system was similar (C statistic, 0.809; 95% CI, 0.756–0.861; optimism-corrected C statistic, 0.808) to that of the logistic regression model at internal bootstrap validation.

**Conclusions:**

This study resulted in the development and internal verification of a scoring system to classify 334 patients at risk for falls. The newly developed score demonstrated greater accuracy in predicting falls in elderly people than did the Timed Up and Go test and the 30-Second Chair Sit-Stand test. Additionally, the scale demonstrated superior clinical validity for identifying fall risk.

**Supplementary Information:**

The online version contains supplementary material available at 10.1186/s12877-024-05064-4.

## Introduction

Falls and fall-related injuries are leading causes of morbidity and mortality in older people [1, 2]. Approximately 30% of people aged more than 65 years experience a fall once every year, and approximately 32,000 deaths among older adults result from fall-related injuries [3–5]. A fall is defined as an event (including syncopal events) that results in a person coming to rest inadvertently on the ground or floor or at another lower level [6]. Given that many falls can be prevented, a short, easy-to-administer, multifactorial fall risk assessment is crucial for developing targeted interventions [7–10].

Hence, several reports have evaluated the accuracy of fall risk assessment tools, which are recommended for elderly people [11, 12]. The self-rated Fall Risk Questionnaire (self-rated FRQ) is a fall risk screening component of the Stopping Elderly Accidents, Deaths, and Injuries (STEADI) toolkit and is widely used in many centers [13, 14]. The Cronbach’s α of the self-rated FRQ was slightly lower (0.670) in Chinese community-dwelling older adults, implying that less irrelevant items need to be removed and more relevant items need to be added to the questionnaire [15]. The FRQ assessment completely depends on patient complaints and lacks objective assessment data; therefore, information bias is likely to occur. In practice, most fall risk assessments are usually collected through patient interviews, questionnaires, and simple physical performance tests. However, these assessments have relatively high false-positive rates when used in isolation. A single tool still faces the problem of one-sided evaluation, which focuses on certain risk factors. Worldwide guidelines for fall prevention and management for older adults propose considering objective factors, such as balance abilities, as well as subjective risk factors, which include the level of concern older adults have about falling [6]. In addition, researchers recommend that fall risk assessment tools should not be used in isolation to identify older people at high risk of falls [16]. Therefore, it is important to explore a quick and reproducible score that covers the subjective history and objective data to ensure that the measured results more truly reflect the patient’s state. Moreover, fall risk levels can be evaluated to facilitate fall prevention management programs for individuals with low, medium and high risk of falls. This study was designed to develop and internally validate a novel risk score to classify patients at risk for falls.

## Data collection methods

A total of 334 individuals aged > 60 years who attended the fall clinic from January 01, 2019, to January 01, 2021, were selected for the questionnaire survey and evaluation. The exclusion criteria were as follows: (1) aged < 60 years; (2) incomplete questionnaire and evaluation data or missing relevant information records; and (3) stroke, Alzheimer’s disease, or other medical diseases that affected activity.

## Outcome measures

The follow-up ended on December 12, 2021. The outcome was whether the participant fell again within one year of the follow-up period. The older individuals were followed up by telephone interviews with designated persons.

## Statistical methods

### Evaluation of predictors and variable selection

Least absolute shrinkage and selection operator (LASSO) regression was used to minimize the potential concatenation of variables measured from the same patient to identify the key variables associated with 1-year falls. We conducted 5-fold cross-validation to screen for the most useful predictive variables using the “glmnet” R package. The absolute magnitude of the coefficients of the regression model was penalized according to the value of λ. The most predictive covariate was selected using the minimum value, lambda.min (λ_min_).

### Development of the prediction model and nomogram construction

We included either the individual items FRQ-Q1 through FRQ-Q12, or the total FRQ score as independent variables for variable selection by employing lasso regression. Subsequently, two logistic regression models were developed to predict 1-year falls using these variables. Model 1 utilized FRQ-Q1 to FRQ-Q12 as predictors, while Model 2 employed the total FRQ score for this purpose. Backward stepwise logistic regression based on the likelihood ratio test with the Akaike information criterion (AIC) were applied to select the optimal models. The nomogram was subsequently drawn using R 4.1.3.

To elucidate the differences in C-statistics between Model 1 and Model 2, we also developed two univariate models for further comparison: Model 3, which utilized the Time Up and Go (TUG) test, and Model 4, which employed the number of chair sit-to-stand tests completed in 30 s.

### Internal validation

The model performances in terms of overall accuracy (e.g., Brier score), discrimination ability (e.g., C statistic), and calibration ability (e.g., calibration curves) were evaluated for internal validation via the bootstrap method (1000 repetitions). For each bootstrap iteration, we implemented a LASSO regression model to select features, followed by fitting a logistic regression model. The clinical usefulness and net benefit were estimated via decision curve analysis. Risk stratification models were also compared using continuous/category net reclassification improvement (NRI) and integrated discrimination improvement (IDI) methods [17].

### Development of the scoring system

A point system to estimate the risk of 1-year falls was developed to simplify the evaluation. We established independent risk factors (i) for falls within a 1-year fall and their regression coefficients (Bi) using the optimal logistic regression model. The risk factors were categorized, and the base category with the lowest risk for each factor was used as the reference value (WiREF, 0 points). Higher-risk categories were assigned progressively increasing reference values (Wij), which reflect increased risk. The reference values for binary variables were assigned 0 for “no” and 1 for “yes.” The continuous variables (e.g., single-leg standing time) were grouped. The median value of each group was used as the reference value. A constant (B) reflecting a 1-point increase in the score was established. The number of points for each categorical change was calculated by dividing the number of regression units for that categorical change (Distance from WiREF) by the constant, after which the results were rounded to the nearest integer.

$$Points= D\left(Distance from WiREF\right)/B\left(constant\right)$$

$$\eqalign{&Estimated\,Risk = \cr \\&\quad{1 \over {\left( {1 + \exp ( - (B(score) + \sum\nolimits_{(i = 1)}^n {(\beta i * WiREF)} + {^\prime}the\,intercept{^\prime}))} \right)}} \cr}$$

The estimate of risk for each point total was calculated using exponentiation of the linear predictor of the optimal model; moreover, the intercept, the total score, and the constant (B) and base values for the continuous risk factors were also considered.

SPSS 25.0 and R 4.1.3 software were used for statistical analysis. The categorical variables were expressed as the frequency and constituent ratio, and the continuous data, which did not follow a normal distribution, were expressed as M (P25–P75). The chi-square test was used for the univariate analysis of qualitative data, and the nonparametric test was used for continuous data.

## Results

### Basic information about the participants

A total of 334 older people were categorized into a fall group (119 people) and a nonfall group (215 people) based on whether they had fallen within 1 year. The basic characteristics of the two groups are shown in eTable 1 (Supplementary Files).

### LASSO regression feature selection

**Fig. 1:**
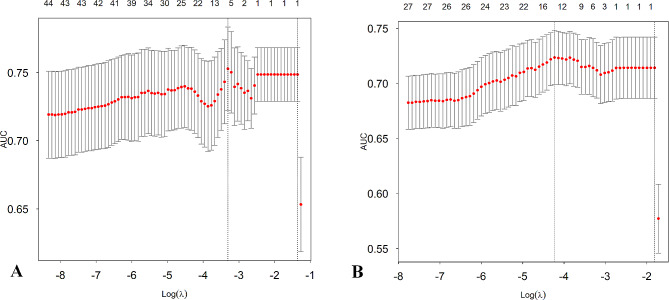
Texture feature selection using the least absolute shrinkage and selection operator (LASSO) binary logistic regression model. **A**, Tuning parameter (λ) selection in the LASSO model used 10-fold cross-validation via minimum criteria. The area under the receiver-operating characteristic curve (AUROC) was plotted versus log(λ) in Model 1. **B**, Tuning parameter (λ) selection in the LASSO model used 10-fold cross-validation via minimum criteria. The AUROC was plotted versus log(λ) in Model 2

Two LASSO regression models were established for variable screening, taking the occurrence of falls within 1 year of follow-up as the dependent variable and the collected indicators as the independent variables. Model 1 included the FRQ-Q1 to FRQ-Q12(First statement of FRQ- twentieth statement of FRQ), whereas Model 2 included the total FRQ score; the remaining variables were the same. The results are shown in Fig. 1. Model 1 included five variables and the λmin: history of hypotension, FRQ-Q1 (I have fallen in the past year), FRQ-Q3 (Sometimes I feel unsteady when I am walking), FRQ-Q5 (I am worried about falling), and single-leg standing duration (left foot); the largest area under the curve (AUC) is shown in Fig. 1A. Model 2 included fifteen variables, namely, sex, BMI, history of hypotension, osteoporosis, fracture, anemia, abnormal vision, abnormal hearing, abnormal foot sensation, use of a walking aid, total FRQ score, time between instep and toe contact (front of the left foot), time between instep and toe contact (front of the right foot), time between heel and toe contact (front of the left foot), and single-leg standing duration (left foot) (Fig. 1B).

### Logistic regression model

The variables selected by LASSO regression were used as independent variables, and falls within 1 year of follow-up was used as the dependent variable in the logistic regression model. Backward stepwise regression was performed based on the likelihood ratio test with the AIC. The results are shown in Table 1. Model 1 corresponded to the FRQ subscore, and Model 2 corresponded to the total FRQ score.

**Table 1:** Logistic regression analysis of fall risk prediction for the study participants

	β	SE	OR (95% CI)	*P*
Model 1				
FRQ-Q1	3.709	0.467	40.790 (16.350–101.800)	< 0.001
FRQ-Q3	0.667	0.295	1.950 (1.090–3.470)	0.024
Single-leg standing time (left foot), s	–0.353	0.146	0.700(0.530–0.940)	0.016
Model 2				
History of hypotension	0.999	0.452	2.710 (1.120–6.580)	0.027
Total FRQ score	0.432	0.063	1.540 (1.360–1.740)	< 0.001
Instep touch the toes (front of left foot), s	–0.207	0.092	0.810 (0.680–0.970)	0.024

Table 1 presents odds ratios associated with fall risk and predictive variables in Model 1 and Model 2 providing coefficient values (β), standard errors (SE), and significance levels (*P*. value). Odds ratios are offered, along with 95% confidence intervals (95% C.I. for Exp (B)) for each model. The significance level is denoted at *p* < 0.05. FRQ-Q1 = I have fallen in the past year; FRQ-Q3= Sometimes I feel unsteady when I am walking; Total FRQ score = The total score of self-rated Fall Risk Questionnaire

**Fig. 2:**
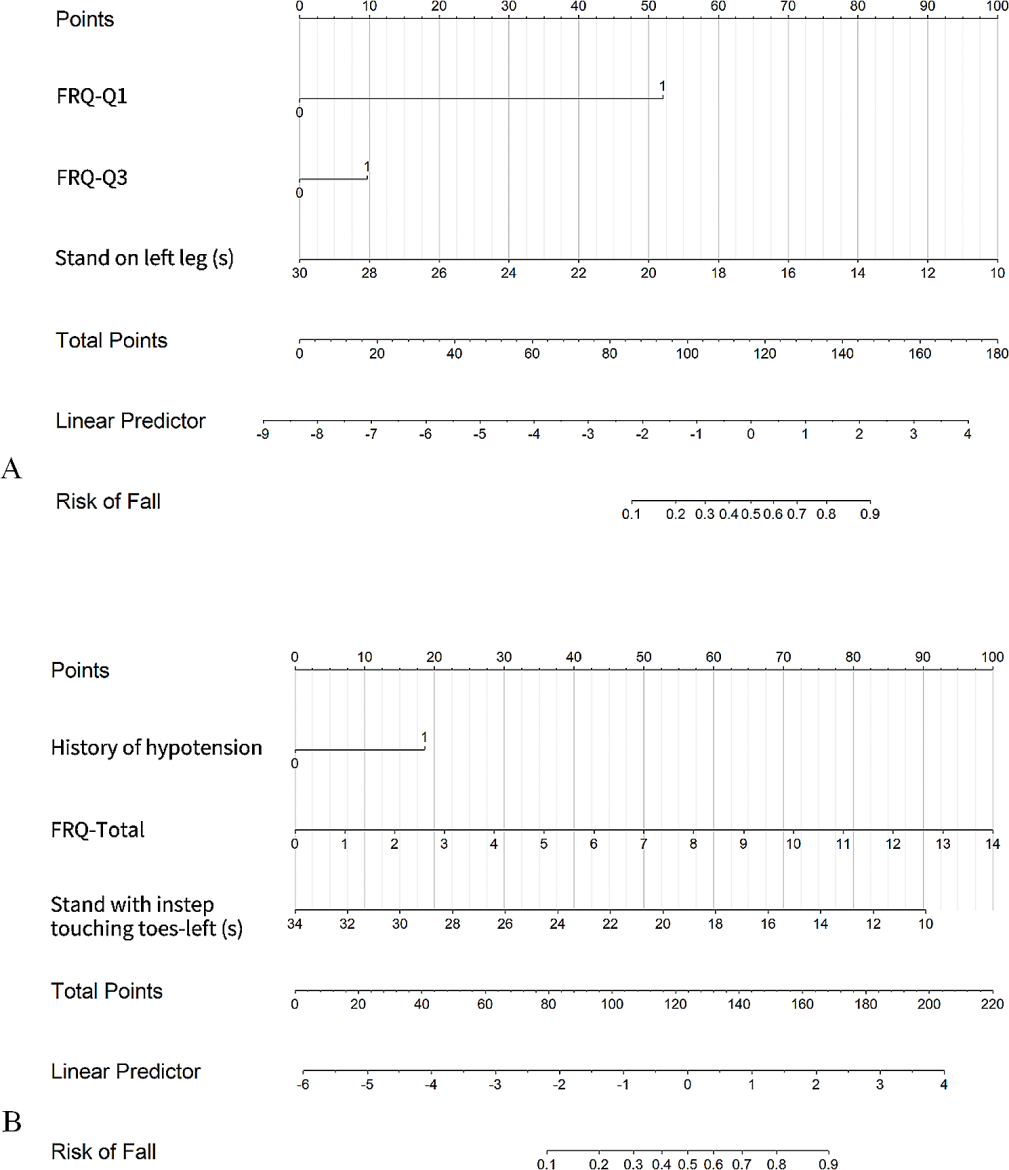
Nomogram for the prediction of falls (**A**) A nomogram was constructed based on the data in Model 1 (**B**) A nomogram was constructed based on the data in Model 2 The points of each feature were added to obtain the total points, and a vertical line was drawn on the total points to obtain the corresponding ‘risk of fall’. FRQ-Q1 = I have fallen in the past year; FRQ-Q3 = Sometimes I feel unsteady when I am walking; FR-Total = The total score of the self-rated Fall Risk Questionnaire

Model 1 included FRQ-Q1, FRQ-Q3, and single-leg standing duration (left foot). Among these factors, FRQ-Q1 and FRQ-Q3 were positively correlated with fall risk, but single-leg standing duration (left foot) time was negatively correlated with fall risk. Model 2 included history of hypotension, total FRQ score, and time between instep and toe contact (the front of the left foot). A history of hypotension and total FRQ score were positively correlated with fall risk, but the duration of contact with the toes (the front of the left foot) was negatively correlated with fall risk. A fall risk prediction nomogram was established for participants based on the logistic regression model. The results are shown in Fig. 2.

### Validation of the fall risk prediction nomogram model

**Fig. 3:**
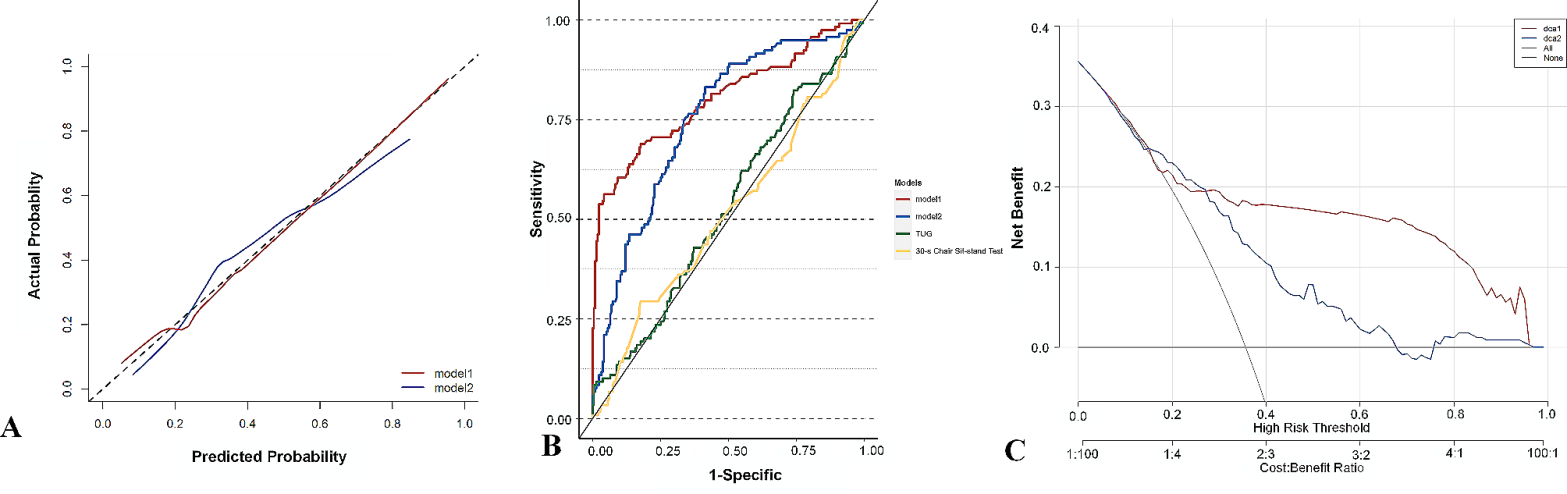
(**A**) Calibration curves of the nomogram prediction in Model 1 and Model 2. (**B**) The test result variable(s) C-statistics of Model 1 (0.803), Model2 (0.752), the TUG test (0.530) and 30s’chair sit-to-stand test were 0.512; C-statistics = Area Under Curve. (**C**) Decision curve analysis (DCA) of the nomogram prediction in Model 1 and Model 2

The bootstrap internal validation method was used for internal validation, and the calibration curve and receiver-operating characteristic (ROC) curve were obtained, as shown in Fig. 3. The abscissa and the ordinate of the calibration curve were used to predict the incidence of the event and the actual occurrence proportion of the event, respectively. The closer the point is to the diagonal dashed line, the better the calibration of the model. Figure 3A shows that the calibration of Model 1 was better. Model 1 showed better overall accuracy (optimism-corrected Brier score) than Model 2 (0.181 vs. 0.230) according to internal bootstrap validation (Table 2). Furthermore, Model 1 also showed better discrimination (Table 2).

**Table 2:** Predictive Performance for the Model1, Model2, and Score System by Development and Internal Validation Settings

Model Performance		Predicting in community-dwelling older people		
		Model1	Model2	Score System
C statistic		0.803 (0.749–0.857)	0.752 (0.698–0.806)	0.809 (0.756–0.861)
*P* value		0.068		NA
IDI		0.205 (0.147–0.262)	Ref.	NA
*P* value		< 0.01		NA
Continuous NRI		0.468 (0.314–0.622)	Ref.	NA
*P* value		< 0.01		NA
Categorical NRI		0.507 (0.291– 0.724)	Ref.	
P value		< 0.01		
*Internal* *Validation*	C statistic (Optimism-corrected)	0.735	0.671	0.808
	Brier score (Optimism-corrected)	0.181	0.230	0.159

IDI: Integrated discrimination improvement; NRI: Net Reclassification Index. The significance level is denoted at *p* < 0.01

C statistics of 0.803 (95% CI 0.749–0.857) and 0.752 (95% CI 0.698–0.806) were obtained for Model 1 and Model 2, respectively. The minimal overfitting (optimism-corrected C statistic) was 0.735 in Model 1 and 0.671 in Model 2 with internal bootstrap validation. The variables in Models 3 and 4 were the time required for the TUG (Time up and go test) and the number of chair sit-to-stand tests in 30s. The C statistic of Models 3 and 4 were close to 0.5, which represented poor classification.

Model 1 performed better than Model 2 for the continuous NRI [0.468 (0.314–0.622)], categorical NRI [0.507 (0.291–0.724); cutoff: 0.200–0.800], and IDI [0.205 (0.147–0.262)]. Decision curve analysis was used to compare the full and simple models. As shown in Fig. 3C, both Models 1 and 2 had net benefits above the extreme reference line; moreover, Model 1 provided better net benefits than Model 2.

**Fig. 4:**
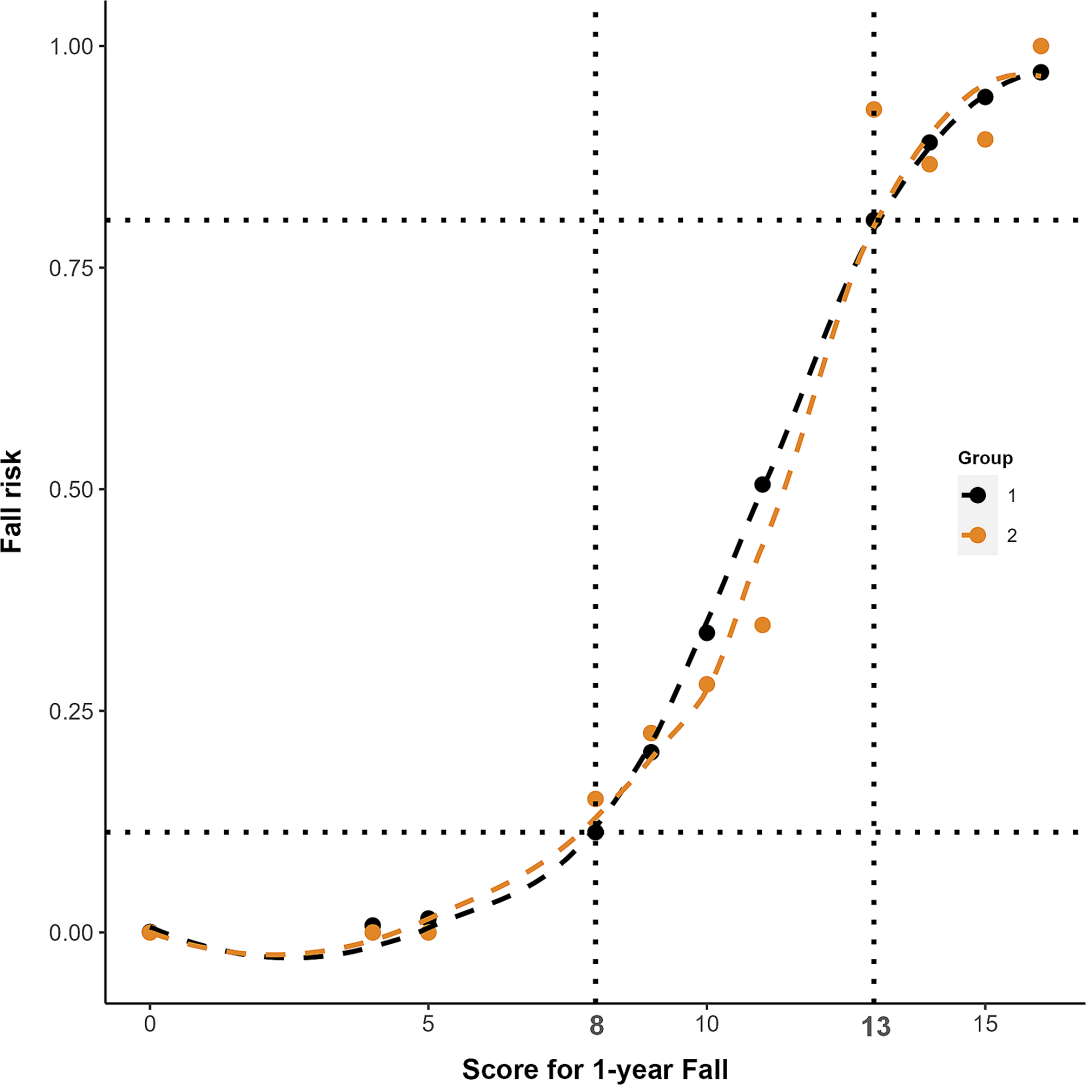
Observed vs. Estimated Fall Risk According to the Numerical Risk Score. 1: Estimated risk; 2: Observed risk

A scoring system was produced based on Model 1 for better predictive performance (Table 3). The associations between the total point score and the predicted mortality are shown in Table 4. The scoring system showed similar discrimination (C statistic, 0.809 95% CI, 0.756–0.861); optimism-corrected C statistic, 0.808) and overall accuracy (optimism-corrected Brier score, 0.159) to those of the logistic regression model at internal bootstrap validation (Table 2). Figure 4 shows the observed and predicted probabilities according to the numerical risk score (Table 3). When the scores were 8 or 13, the observed and predicted risk both apparently increased. Thus, we categorized the fall scores into three groups: low risk (scores 0–7), medium risk (scores 8–12), and high risk (scores 13–19).

**Table 3:** Scoring table for the new model

Predictors	Categories	Reference value	Beta	D	Points
Single leg (left foot)			–0.357		
	26–30	28		0	0
	21–25	23		1.787	3
	16–20	18		3.574	5
	11–15	13		5.361	8
	6–10	8		7.148	10
	0–5	2.5		9.114	13
FRQ-Q1			3.720		
	No	0		0	0
	Yes	1		3.720	5
FRQ-Q3			0.694		
	No	0		0	0
	Yes	1		0.694	1

Beta = Regression coefficients, D = Distance from WiREF

**Table 4:** Total point and estimated risk table

Total point	Estimated risk	Total point	Estimated risk
0	0.0005	10	0.3380
1	0.0010	11	0.5054
2	0.0020	12	0.6716
3	0.0040	13	0.8036
4	0.0079	14	0.8912
5	0.0157	15	0.9425
6	0.0309	16	0.9704
7	0.0599	17	0.9850
8	0.1131	18	0.9924
9	0.2033	19	0.9962

## Discussion

In this study, we developed and internally validated a simple, fast-screening and reproducible fall risk assessment model that covers subjective history and objective data to assess fall risk in healthy older adults living in the community. The potential risk factors for falls were integrated through logistic regression analysis. We assigned a score to the level of each risk factor based on its impact on the risk of falling and subsequently summed all the scores to derive a total score. Finally, the risk of falling was calculated using the total score. That score could not only predict the probability of falls individually and accurately but could also summarize all the results on a scoring axis, helping clinicians obtain information more quickly and intuitively. The novel model revealed a C statistic of 0.803, which indicates that 80.3% of the older persons were classified correctly by using this score. Additionally, we used other fall risk assessment tools, including the TUG and 30-s chair sit-stand test score, but the C-statistic value of the TUG and 30-s chair sit-stand test score was close to 0.5, indicating poor classification. These results are consistent with those of other researchers [18]. Fall risk assessment tools should not be used in isolation to identify older people at high risk of falls.

Single-leg standing time was the strongest predictor of fall risk in the new model consisting of three simple predictors. Moreover, single-leg standing time is a method of quantifying static balance ability that has low requirements in terms of space, equipment, resources, time, familiarity and training. A growing number of studies have demonstrated that this approach should be implemented in primary care to help identify individuals at greater risk of falling [19–22]. Falls were directly associated with balance test abnormalities. Research has shown that people who cannot complete a 5-s single-leg standing have a 2.2 times greater risk of falling than do those who can [23]. Moreover, Muir reported that people with single-leg standing times greater than 10 s have a 1.58-fold greater risk of falling after a 12-month follow-up [24]. The evaluation of balance with the single-leg standing test is a logical and functional approach since transient balancing of a single limb is essential for individuals with a normal gait and is critical for activities of daily living, such as turning, stair climbing, and dressing [25].

Several studies have shown that balance training programs reduce the risk of falls in elderly individuals [26–29]. Compared with several intrinsic factors, such as age, balance function can be improved by exercise intervention [8]. Moreover, the single-leg standing test has been found to be associated with the risk of all-cause death in middle-aged and older people who cannot complete the 10-s single-leg standing test; these individuals have a greater risk of all-cause death and a shorter life expectancy [30–32].

The single-leg standing test has been proven to be a challenging test that can be used to screen people for falls < 1.02 s, with a sensitivity of 0.67 and a specificity of 0.89 [33]. However, single-leg standing times are not recommended as a stand-alone indicator for fall risk screening because of their high specificity and rather low sensitivity. Recently, it has been suggested that at least two screening tools should be used together to maximize the advantages of each for predicting the occurrence of falls [34, 35].

According to this new assessment model, in addition to single-leg standing times (left foot), fall history contributed the most to fall risk prediction, followed by the feeling of walking unsteadily at times. Studies show that older people with a history of falls often reduce their activities because of fear of falling [36, 37]. Older people who are highly concerned about falls and who are restricted from daily living activities may even walk less than 1.2 h a day, which may eventually lead to further declines in physical function and falls [38, 39].

This study showed that the new prediction model, the calibration curve, and the standard line were highly consistent. The results suggested that the fall risk predicted using the model was highly consistent with the actual fall risk. A history of falls together with balance or gait disturbances was considered a strong predictor of falls. Therefore, the new model might improve discrimination between individuals who fall and those who do not fall. For better clinical application of the new prediction model, the scoring system was produced based on Model 1, which showed similar discrimination to that of the logistic regression model at internal bootstrap validation. In the new scoring system, single-leg standing times were assigned a score of five points. Most studies recorded a cutoff point of 5 s for one-leg standing [22], and the most common length of the continuous trials was 30 s [4]. This might be due to a rapid decrease in force variability amplitude, as the subjects made postural adjustments to regain standing balance after transferring weight to a single leg. Moreover, the change in the force amplitude occurred within the first 5s of testing. Furthermore, the novel scoring system can be used to evaluate fall risk levels to facilitate fall prevention management programs for individuals with low, medium and high risk of falls. According to risk stratification, a person-centered approach to designing an individualized intervention was recommended. The ‘low risk’ group should be reassessed annually. Older adults in the ‘intermediate risk’ group should perform strength and balance exercise interventions since evidence shows that this type of exercise is effective at reducing fall risk [40]. Finally, a comprehensive fall risk assessment should be offered for those in the ‘high risk’ group.

## Conclusions

A score for predicting the fall risk of elderly people was developed and internally verified; this score exhibited greater accuracy in predicting falls than traditional assessments such as the Timed Up and Go test and the 30-Second Chair Sit-Stand test scores. Additionally, the scale demonstrated superior clinical validity for identifying fall risk.

### Strengths and limitations

A simple scoring system for predicting the risk of falls was developed that could help accurately identify older patients at risk of falls. Fall risk levels can be evaluated to facilitate the development of fall prevention management programs for individuals at low, medium and high risk of falls. The main limitations of this study were that the sample size was small and that the nomogram model was only internally validated. Multicenter and large-sample studies are needed for external validation to reduce bias and to continuously calibrate the model in clinical practice. Furthermore, using existing data to train multiple prediction models (neural network, gradient boosting, support vector machine, and decision forest), we can analyze the difference in prediction accuracy between models, find the optimal solution of data modeling in the current dimension, and avoid overfitting and underfitting. The strong adaptability of the model will be ensured when it is applied to the real world.

### Electronic supplementary material

Below is the link to the electronic supplementary material.

Supplementary Material 1

## Data Availability

The original contributions presented in this study are included in the article/supplementary material, and further inquiries can be directed to the corresponding author.
