# Supplementary material for: A novel score for predicting falls in community-dwelling older people: a derivation and validation study
Source: BMC Geriatr. 2024 Jun 4;24:491. doi: 10.1186/s12877-024-05064-4 (PMC11151514; doi:10.1186/s12877-024-05064-4)
Supplement: Supplementary file 1 — Supplementary Material 1 [file 12877_2024_5064_MOESM1_ESM.docx]

Supplementary Appendix

This appendix has been provided by the authors to give readers additional information about their work.

Supplemental Appendix: A novel score for predicting falls in community-dwelling older people: A derivation and validation study.

Ming Zhou^1†^, Gongzi Zhang^1†^, Na Wang^1^,Tianshu Zhao^1,3^, Yangxiaoxue Liu^1,3^,Yuhan Geng^1,3^,Ning Wang^1^,Nan Peng^2*^,Liping Huang^1*^

^†^Contributed equally ^*^Correspondening author

**eTable 1.** Basic information and assessment results of the fall and non-fall groups

| Predictors | Total (n = 334 ) | Fall group (n = 119) | Non-fall group(n = 215 ) | P |
| --- | --- | --- | --- | --- |
| Age, years | 69.38 ± 6.25 | 69.45 ± 6.44 | 69.34 ± 6.16 | 0.88 |
| BMI, kg/m2 | 23.7 ± 2.95 | 23.55 ± 3.06 | 23.79 ± 2.88 | 0.49 |
| Female | 218 (65.3) | 89 (74.8) | 129 (60.0) | 0.007 |
| Hypertension | 130 (38.9) | 49(41.2) | 81(37.7) | 0.53 |
| Hypotension | 27 (8.1) | 16(13.4) | 11(5.1) | 0.007 |
| Diabetes | 35 (10.5) | 13(10.9) | 22(10.2) | 0.84 |
| Neurological disorders | 46 (13.8) | 20(6.0) | 26(7.8) | 0.23 |
| Osteoporosis | 136 (40.7) | 56(47.1) | 80(37.2) | 0.07 |
| Joint degeneration | 165 (49.4) | 64((53.8) | 101(47.0) | 0.23 |
| Fractures | 14 (4.2) | 9(7.6) | 5(2.3) | 0.045 |
| Urinary incontinence | 45 (13.5) | 21(17.6) | 24(11.2) | 0.096 |
| Anemia | 7 (2.1) | 2(1.7) | 5(2.3) | 0.694 |
| Visual abnormalities | 258 (77.2) | 93(78.2) | 165(76.7) | 0.769 |
| Hearing abnormalities | 254 (76.0) | 94(79.0) | 160(74.4) | 0.348 |
| Paresthesias in the feet | 115 (34.4) | 40(33.6) | 75(34.9) | 0.815 |
| Use of a walker | 8 (2.4) | 4(3.4) | 4(1.9) | 0.627 |
| Recumbent SBP, mm Hg | 131.57 ± 17.6 | 130.91 ± 16.31 | 131.93 ± 18.31 | 0.61 |
| Recumbent DBP, mm Hg | 73.17 ± 8.56 | 72.54 ± 8.74 | 73.52 ± 8.46 | 0.32 |
| Recumbent pulse, times | 70.97 ± 9.25 | 72.03 ± 9.33 | 70.38 ± 9.17 | 0.12 |
| Standing SBP, mm Hg | 128.37 ± 18.76 | 126.94 ± 17.4 | 129.15 ± 19.47 | 0.30 |
| Standing DBP, mm Hg | 73.5 ± 9.71 | 72.82 ± 9.54 | 73.87 ± 9.8 | 0.35 |
| Standing pulse, times | 73.37 ± 9.96 | 74.37 ± 10.16 | 72.81 ± 9.82 | 0.17 |
| Blood oxygen saturation | 96.61 ± 2.95 | 96.45 ± 3.12 | 96.71 ± 2.85 | 0.44 |
| Fsbt-1, s | 11.76 ± 2.38 | 11.62 ± 2.31 | 11.83 ± 2.42 | 0.43 |
| Fsbt-2 (left foot), s | 11.73 ± 2.28 | 11.49 ± 1.82 | 11.86 ± 2.49 | 0.15 |
| Fsbt-2 (right foot), s | 11.77 ± 2.48 | 11.41 ± 1.42 | 11.97 ± 2.88 | 0.02 |
| Fsbt-3 (left foot), s | 11.75 ± 2.15 | 11.81 ± 2.04 | 11.72 ± 2.2 | 0.72 |
| Fsbt-3 (right foot), s | 11.74 ± 1.91 | 11.64 ± 1.61 | 11.8 ± 2.07 | 0.47 |
| Fsbt-4 (left foot), s | 11.37 ± 1.56 | 11.21 ± 1.05 | 11.46 ± 1.77 | 0.15 |
| Fsbt-4 (right foot), s | 11.09 ± 2.76 | 10.85 ± 2.23 | 11.22 ± 3.01 | 0.25 |
| TUGT, s | 8.85 ± 2.42 | 9.14 ± 2.82 | 8.69 ± 2.15 | 0.10 |
| 30-s chair sitting up test | 15.24 ± 5.34 | 15.2 ± 5.27 | 15.26 ± 5.38 | 0.93 |
| FRQ-Q1 | 71 (21.3%) | 64 (53.8%) | 7 (3.3%) | <0.000 |
| FRQ-Q2 | 14 (4.2%) | 8 (6.7%) | 6 (2.8%) | 0.152 |
| FRQ-Q3 | 125 (37.4%) | 60 (50.4%) | 65 (30.2%) | <0.000 |
| FRQ-Q4 | 37 (11.1%) | 19 (16.0%) | 18 (8.4%) | 0.034 |
| FRQ-Q5 | 174 (52.1%) | 79 (66.4%) | 95 (44.2%) | <0.000 |
| FRQ-Q6 | 97 (29.0%) | 43 (36.1%) | 54 (25.1%) | 0.034 |
| FRQ-Q7 | 47 (14.1%) | 20 (16.8%) | 27 (12.6%) | 0.285 |
| FRQ-Q8 | 105 (31.4%) | 42 (35.3%) | 63 (29.3%) | 0.259 |
| FRQ-Q9 | 156 (46.7%) | 63 (52.9%) | 93 (43.3%) | 0.089 |
| FRQ-Q10 | 60 (18.0%) | 24 (20.2%) | 36 (16.7%) | 0.435 |
| FRQ-Q11 | 81 (24.3%) | 30 (25.2%) | 51 (15.3%) | 0.761 |
| FRQ-Q12 | 90 (26.9%) | 38 (31.9%) | 52 (24.2%) | 0.126 |
| Total FRQ score | 3.42 ± 2.59 | 4.72 ± 2.7 | 2.7 ± 2.23 | <0.000 |

In the Table 1 BMI= Body mass index , DBP=Diastolic blood pressure, SBP= Systolic blood pressure ,TUG=Time Up and Go, FRQ=Fall risk questionnaire, Fsbt = Four-stage balance test, Fsbt-1= Stand with your feet side-by-side, Fsbt-2= Place the instep of one foot so it is touching the big toe of the other foot, Fsbt-3= Tandem stand: Place one foot in front of the other, heel touching toe, Fsbt-4= Stand on one foot, FRQ-Q1 to FRQ-Q12= the subscore of FRQ. P. value<0.05 indicates significant relationship between assessment results of the fall and non-fall gro
